# Role of B1 antisense RNA on the proliferation and killing tumor ability of aged mouse spleen lymphocytes

**DOI:** 10.1038/s41598-025-23139-z

**Published:** 2025-11-07

**Authors:** Xiaodie Wang, Luqman Ali, Wenxia Wang, Yuecheng Yang, Chongguang Wu, Guozhong Zhang, Xu Feng, Yu Wang, Hanwen Zhang, Run Wang, Kai Zhang, Zhanjun Lv, Xiufang Wang

**Affiliations:** 1 Department of Genetics, Hebei Key Lab of Laboratory Animal, Hebei Medical University, 361 East Zhongshan Road, Shijiazhuang, 050017 Hebei People’s Republic of China; 2 A401 Haihe Laboratory of Synthetic Biology, Tianjin ChunYao Biotechnology Limited Company, No. 21 West 15th Avenue, Tianjin Airport Economic Area, Tianjin, 300000 People’s Republic of China

**Keywords:** Mouse B1 antisense RNA, Aged mice, Spleen lymphocytes, Zinc finger protein 92, Short interspersed nuclear elements, Cancer immunotherapy, Drug development

## Abstract

To explore whether mouse short interspersed nuclear element B1 antisense RNA (B1 antisense RNA, B1 asRNA) could improve the proliferation activity and killing tumor function of spleen lymphocytes from aged mice and to investigate the underlying mechanisms, we transfected B1 asRNA into spleen lymphocytes isolated from 12-month-old mice. We found that B1 asRNA substantially increased the proliferative rate of lymphocytes, the number of EdU-positive cells and the number of S phase cells. B1 asRNA decreased the apoptosis of lymphocytes and regulated the mRNA expression levels of senescence-related genes and transcription factor genes. B1 asRNA enhanced the ability of killing S180 and H22 tumor cells of lymphocytes. The immunofluorescence results showed that B1 asRNA increased the fluorescence intensity of ZFP92 protein in the nuclei, and the ChIP-qPCR results indicated that B1 asRNA promoted the binding of ZFP92 protein to DNA sequences of Nanog, Oct4, Sox2, Klf4 and Myc genes. We believe that B1 asRNA regulates the expression of senescence-related genes, cell cycle genes and transcription factor genes and that ZFP92 protein may play an important role in the process of B1 asRNA regulating gene expression. These studies suggest that B1 asRNA can enhance immune functions of senescent lymphocytes.

**Supplementary Information:**

The online version contains supplementary material available at 10.1038/s41598-025-23139-z.

## Introduction

Lymphocytes are important cellular component of organism’s immune function. The spleen contains a large number of macrophages and lymphocytes that participate in the cellular and humoral immunity of the body^1,2^. Lymphocyte dysfunction occurs in the process of aging, which increases susceptibility to diseases such as infectious diseases and cancer^3–5^. How to delay the life span of lymphocytes in aged individuals and to improve their immune function is a scientific problem that has yet to be solved.

Our previous works showed that short interspersed nuclear elements (SINEs) including human Alu and mouse B1 elements have important effects on cell proliferation and other properties in specific environments^6^. The discovery that SINE elements can be affected and even reverse cellular senescence confirms that SINE elements can be targeted to slow cellular senescence^7^. Transposable elements (TEs) account for about 45% of the human genome^8^ and for 37% of the mouse genome^9^. TEs can be used as cis-regulatory elements to participate in gene expression regulation. They are divided into two main types of elements: retrotransposons and DNA transposons. Retrotransposons are further divided into long terminal repeat, long interspersed nuclear elements and SINEs^10^. These TEs are widely distributed in the genomes of eukaryotes and play an important role in genome complexity, gene passivation, generation of new genes, and especially regulation of gene expression^11^.

Primate Alu and mouse B1 families are derived from 7SL RNA and contain RNA polymerase III dependent transcriptional promoters^12^. When cells are exposed to oxidative stress, infection with viruses, or are in a senescent state, genomic epigenetic markers (such as methylation) are unstable, thereby activating SINE transcription^13,14^. In patients with geographical atrophy, Alu RNA expression increases in retinal pigment epithelial cells, which induces cell degeneration and aggravates the occurrence of age-related macular degeneration. Therefore, the antisense RNA of Alu/B1 can be used to inhibit the expression of disease-related genes by targeting and complementing Alu/B1 RNA, thereby preventing SINE RNA from driving the occurrence of age-related diseases^13,15^.

Recently, it has been reported that the SINE sequence in the genome is related to the Kruppel-associated box domain-containing zinc-finger proteins (KRAB-ZFP), such as ZFP92. ZFP92 interacts with SINE elements to regulate chromatin accessibility and effectively changes cellular properties^16^. However, whether SINE antisense RNA affects the action of ZFP92 with SINEs has not been studied in detail.

In this study, spleen lymphocytes were isolated from aged BALB/c mice (12 to 16 months). B1 antisense RNA (B1 asRNA) was transfected into the lymphocytes. We found that B1 asRNA can enhance the proliferation viability and the ability of killing tumor cells by lymphocytes, and regulates the binding ZFP92 with DNAs.

## Methods and materials

### Mouse spleen lymphocytes Preparation and transfection

All experimental procedures conducted with animals were approved by the Laboratory Animal Ethical and Welfare Committee of Hebei Medical University (IACUC-Hebmu-2021009). All methods were performed in accordance with relevant guidelines/regulations and all animal practices were performed with full consideration of animal welfare and in accordance with ARRIVE guidelines and regulations.

BALB/c mice were obtained from the Department of Laboratory Animal Science at Hebei Medical University. The spleens of the mice were taken out after BALB/c mice were euthanized by using a 30% volume per minute displacement rate of 100% CO₂. To study the roles of B1 asRNA on lymphocytes, mouse spleen lymphocytes were separated according to a technique previously described^17^. The lymphocytes were adjusted to 4 × 10^6^/ml using RPMI1640 (Eurobio, France) supplemented with 10% FCS (1000U/ml rIL-2), and treated with 10 µg/ml phytohemagglutinin (PHA) (PHA treatment). 45 µl of lymphocyte suspension was stained with 5 µl 0.4% trypan blue (Solarbio, Beijing) according to the instructions of the reagent and the number of lymphocytes was counted under the microscope to detect the survival rate of lymphocytes. The lymphocytes with a viability rate of 95% can be used for further experiments.

250 µl of the above lymphocyte suspension was added into one well of 48-well plates to do next experiments. B1 asRNA was transfected into lymphocytes at 24 h after PHA stimulation according to our published paper (B1 asRNA group)^18^. B1 asRNA purchased from Tianjin ChunYao Biotech Co., Ltd. Briefly, 50 µl of 1 mg/mL B1 asRNA was added into the EP tube, and then 100 µL of 0.1 M CaCl_2_ was added and the mixture was well mixed to form RNA-CaCl_2_ solution. Slowly and drop by drop 50 µl of 2×HBS was added to the RNA-CaCl_2_ solution to form the calcium phosphate transfection (CPT) solution for B1 asRNA and the above transfection mixture was set as 100%. To obtain 2.8% B1 asRNA, 7 µ l of the 100% transfection mixture was added to one well of the 48-well plate (one well contained 243 µ l of 10% FCS-RPMI1640 medium (1000 U rIL-2)). The same method was used to obtain 2.8% Yeast tRNA and 2.8% LacZ3F3R RNA. Yeast tRNA (tRNA) was an RNA control and its base number was 76. Genetically engineered LacZ3F3R (a fragment of the LacZ gene) RNA was used as another control RNA^7^. The sequence of the B1 asRNA was TTTTTTTTTTCGAGACAGGGTTTCTCTGTGTAGCCCTGGCTGTCCTGGAACTCACTNTGTAGACCAGGCTGGCCTCGAACTCAGAAATCCGCCTGCCTCTGCCTCCCAAGTGCTGGGATTAAAGGCGTGCGCCACCATGCCCGGC. The transfected lymphocytes were cultured at 37˚C with 5% CO_2_. After culturing the lymphocytes for 3 d, 250 µl fresh 10% FCS-1640 (1000 U rIL-2/ml) medium was added into one well, and after another 3 d, the original medium was replaced with 250 µl of fresh 10% FCS-1640 (1000 U rIL-2/ml) medium. The above procedures were repeated until the 15 d. During the culture process, the cells were performed a 1: 2 passage when the cells reached 100% confluence.

Next, we collected the lymphocytes for the cell proliferation viability, cell cycle detection, cell apoptosis assays, RT-qPCR and Western blotting.

ZFP92 silencing in the lymphocytes was achieved by transfecting the ZFP92 siRNA (RiboBio, China) into senescent lymphocytes transfected with B1 asRNA. To transiently transfect the lymphocytes with ZFP92 siRNA, ribo*FECT*™ CP reagent was used. ZFP92-specific siRNA oligonucleotides were synthesized according to the following sequences: siZFP92#1, positive-sense strand and antisense strand were 5′- GGAGGCUUCUGGACCUCAATT − 3′ and 5′-UUGAGGUCCAGAAGCCUCCTT-3′; siZFP92#2, positive-sense strand and antisense strand were 5′- CGCGGAUAAGCGAUAUCUATT-3′ and 5′-UAGAUAUCGCUUAUCCGCGTT-3′; siZFP92#3, positive-sense strand and antisense strand were 5′-GGCAAGGCAUUCAGCCGAATT-3′ and 5′-UUCGGCUGAAUGCCUUGCCTT-3′.

### Cell proliferation viability assays using CCK-8 and edu methods

CCK-8 (Wuhan Boster Biological Technology, China) assay was used to assess lymphocyte proliferation based on previously published methods^19^. DNA synthesis was determined by 5-ethynyl-2’-deoxyuridine (EdU) proliferation assay (Beijing Applygen Co. Ltd., China) according to the manufacturer’s instruction^20,21^.

### Flow cytometry

The cell cycle of lymphocytes was detected using a cell cycle assay kit (Suzhou Uelandy Biotechnology Co. Ltd, China) according to the manufacture’s instruction and 20,000 cells were counted using flow cytometry (Becton, Diekinson and Company, USA). Flowjo software (Beckman Coulter, USA) was used to analyze the results. The cell apoptosis was quantified using Annexin V-FITC/PI double staining apoptosis detection kit (Beijing Sevenbio Biotechnology Co. Ltd, China)^22^. Fluorescence was measured by flow cytometry and FITC and PI-PE signals were collected. The results were analyzed using the Accuri C6 software (Becton, Diekinson and Company, USA).

### Detection of lymphocyte cytotoxicity in vitro and in vivo

In vitro experiment, the lactate dehydrogenase (LDH) release assay (Shanghai Beyotime Biotechnology Co. Ltd) was used to detect the killing function of lymphocytes on S180 and H22 tumor cells according to previously published methods^23^. After B1 asRNA treatment, the cytotoxicity of lymphocytes in the PHA group and the B1 asRNA group was determined by LDH release analysis. The ratios of effector cells and target cells were set as 50:1 and 100:1.

The following method was used to explore the killing tumor ability of lymphocytes in vivo: Spleen lymphocytes from aged BALB/c mice were prepared at a concentration of 4 × 10^6^/ml and placed into 24-well plates at 0.5 ml per well. The culture medium was 10% FCS-1640 (1000 U rIL-2), and the cells were divided into a PHA treatment group (PHA group) and B1 asRNA transfection group after based on the PHA treatment (B1 asRNA group). At 6 d after B1 asRNA treatment, the lymphocytes treated by B1 asRNA from 30 wells were mixed, then were made into 3 ml with NS (total cell number in 3 ml was 10.74 × 10^7^). 0.5 ml of cell suspension was injected intraperitoneally into each S180 tumor-bearing mouse and total 6 mice were injected as B1 asRNA group. Similarly, the lymphocytes from 30 wells of the PHA group were collected and made into 3 ml (total cell number in 3 ml was 7.26 × 10^7^), and injected into 6 S180 tumor-bearing mice as PHA group. We collected the lymphocytes at 6 days after B1 asRNA in view of the activating and their continued proliferation capacity of lymphocytes.

S180 cells were inoculated subcutaneously in the right axilla of aged BALB/c mice (≥12 months) to establish the S180 tumor-bearing mouse. On the second day after modeling, lymphocytes were injected into S180 tumor-bearing mice by intraperitoneal injection.

At 15 d after the lymphocytes were injected into S180 tumor-bearing mice, the mice were euthanized by using a 30% volume per minute displacement rate of 100% CO₂ and the tumor was dissected and weighed. During the process of observing the tumor growth, the maximum diameter of the tumor did not exceed 15 mm.

### RT-qPCR analysis of mRNA expression

RT-qPCR was performed according to our previous studies^18^. Table 1 shows the primers for RT-qPCR. The spleen lymphocytes isolated from aged BALB/c mice were seeded in 48-well plates and cultured for 24 h. Subsequently, the cells were treated with 2.8% B1 asRNA for 24, 48–72 h. To analyze the mRNA expression, TRIzol (Thermo Fisher Scientific, Inc.) was used to extract the total RNA from the lymphocytes. Total RNA was reverse-transcribed into cDNA using RevertAid First Strand cDNA Synthesis kit (Thermo Fisher Scientific, Inc.) according to the manufacturer’s protocol. Expression levels of p53, H2AX, Lamin B1, p16 ^Ink4a^, p21, ILα, IL1β, IL6, IL8, MMP3, MMP13, Nanog, Oct4, Sox2, Klf4, c-Myc and c-Fos were detected using qPCR kit (GeneCopoeia, USA). qPCR was performed using the following thermocycling conditions: Initial denaturation at 95˚C for 2 min, followed by 40 cycles at 95˚C for 10 s and 56˚C for 40 s. The 2^−∆∆Cq^ method was used to calculate the relative mRNA expression levels^29^. The primer pairs utilized for RT-qPCR analysis are shown in Table 1.

**Table 1:** RT-qPCR primer used for amplification.

Target	Sequences	Product length (bp)
P53	Forward: 5ʹ-TCACAGTCGGATATCAGCCT-3ʹ	172
	Reverse: 5ʹ-ACACTCGGAGGGCTTCACTT-3ʹ	
H_2_AX	Forward: 5ʹ-AGCGACTCAACTACAACCCAAACA-3ʹ	70
	Reverse: 5ʹ-AGGCTCAGTCCAGACAGGGATT-3ʹ	
P21	Forward: 5ʹ-ACTTCCTCTGCCCTGCTGC-3ʹ	141
	Reverse: 5ʹ-GCTGGTCTGCCTCCGTTTT-3ʹ	
p16^Ink4a^	Forward: 5′-CAGGTGATGATGATGGGCAACG-3′	146
	Reverse: 5′-TGCAGCACCACCAGCGTGTC-3′	
Sirt1	Forward: 5ʹ-GCCACCAACACCTCTTCATA-3ʹ	233
	Reverse: 5ʹ-TACTGGAACCAACAGCCTTA-3ʹ	
Lamin B1	Forward: 5ʹ-GGGAAGTTTATTCGCTTGAAGA-3ʹ	62
	Reverse: 5ʹ-ATCTCCCAGCCTCCCATT-3ʹ	
c-Fos	Forward: 5ʹ-GGGACAGCCTTTCCTACTACC-3ʹ	88
	Reverse: 5ʹ-AGATCTGCGCAAAAGTCCTG-3ʹ	
Nanog	Forward: 5ʹ-GCAGAAGTACCTCAGCCTCC-3ʹ	110
	Reverse: 5ʹ-ACCGCTTGCACTTCATCCTT-3ʹ	
Myc	Forward: 5ʹ-TGACCTAACTCGAGGAGGAGCTGGAATC-3ʹ	170
	Reverse: 5ʹ-AAGTTTGAGGCAGTTAAAATTATGGCTGAAGC-3ʹ	
Oct4	Forward: 5ʹ -TGTTCAGCCAGACCACCATC-3ʹ	100
	Reverse: 5ʹ-GCTTCCTCCACCCACTTCTC-3ʹ	
Sox2	Forward: 5ʹ-AGGAGAGAAGTTTGGAGCCC-3ʹ	152
	Reverse: 5ʹ-TCTGGCGGAGAATAGTTGGG-3ʹ	
Klf4	Forward: 5ʹ-GTGCCCCGACTAACCGTTG-3ʹ	185
	Reverse: 5ʹ-GTCGTTGAACTCCTCGGTCT-3ʹ	
IL1α	Forward: 5′-AGGAGAGCCGGGTGACAGTA -3′	51
	Reverse: 5′-TCAGAATCTTCCCGTTGCTTG -3′	
IL1β	Forward: 5′-CCAAAAGATGAAGGGCTGCT-3′	51
	Reverse: 5′-TCATCAGGACAGCCCAGGTC-3′	
IL6	Forward: 5′-CTGCAAGAGACTTCCATCCAG-3′	131
	Reverse: 5′-AGTGGTAGACAGGTCTGTTGG -3′	
IL8	Forward: 5′-CTGGTCCATGCTCCTGCTG-3′	51
	Reverse: 5′-GGACGGACGAAGATGCCTAG-3′	
MMP3	Forward: 5′-ACTCCCTGGGACTCTACCAC-3′	163
	Reverse: 5′-GGTACCACGAGGACATCAGG-3′	
MMP13	Forward: 5′-GATGGACCTTCTGGTCTTCT -3′	136
	Reverse: 5′-GCTCATGGGCAGCAACAATA -3′	
β⁃actin	Forward: 5ʹ-CTGTGCCCATCTACGAGGGCTAT-3ʹ	155
	Reverse: 5ʹ-TTTGATGTCACGCACGATTTCC-3ʹ	

### Western blotting assay

The spleen lymphocytes isolated from aged BALB/c micewere seeded in 48-well plates and cultured for 24 h. Subsequently, the cells were treated with 2.8% B1 asRNA for 24,48–72 h. Then, the cells were lysed with RIPA buffer (Cell Signaling Technology, Inc.). Protein levels were measured with the BCA protein assay kit (Beijing Dingguo Changsheng Biotechnology Co., Ltd., China). Protein samples, each containing 40 µg per lane, were denatured and then analyzed using 12.5% SDS-PAGE electrophoresis, and transferred to a nitrocellulose membrane (Pall Corporation, USA). The membrane was blocked and then incubated overnight with an anti-Nanog (cat. no.WL03273), anti-Oct4 (cat. no. WL02020), anti-Sox2 (cat. no.WL03767), anti-Klf4 (cat. no. WL02600), anti-Myc (cat. no. WL01781) or anti-c-Fos (cat. no. WL03699) IgG (Shenyang Wanleibio Biotechnology, China). Anti-β-actin IgG (cat. no. WL01372, Shenyang Wanleibio Biotechnology, China) was served as a loading control. After incubation with a horseradish peroxidase-conjugated anti-IgG antibody (cat. no. WLA023, Shenyang Wanleibio Biotechnology, China), the blots were developed with the ECL kit (Wanleibio Biotechnology Co. Ltd., China) and visualized using a chemiluminescence imaging system (Bio-Rad Biotechnology Co., Ltd., USA). The intensity of each band was normalized to that of β-actin. Gel-Pro analyzer software (Media Cybernetics, L.P., USA) was used to analyze the integrated optical density (IOD) value of each lane.

### Immunofluorescence

To evaluate the expression levels and distribution of ZFP92 protein, lymphocytes were fixed with 4% paraformaldehyde for 15 min. The ZFP92 antibody was diluted 500:1 with sealing solution and was incubated overnight at 4 ˚C. FITC-labeled sheep anti-rabbit antibodies were diluted 500:1 using a sealing solution. Cells were incubated with 1 µg/mL DAPI (Shaihai Yeasen Biotechnology Co. Ltd., China) away from light for 5 min and then were observed under a fluorescence microscope (Olympus Company, Japan).

### Chromatin immunoprecipitation (ChIP)-qPCR

ChIP-qPCR was performed using a SimpleChIP^®^ Plus Enzymatic Chromatin IP Kit (Cell Signaling Technology, Inc., USA) according to the instructions of the kit. Briefly, to investigate the effect of mouse B1 asRNA on the binding of ZFP92 protein with transcription factors Nanog, Oct4, Sox2, Klf4 and Myc DNA in spleen lymphocytes, the conventional cultured lymphocytes were used as the control group, and the B1 asRNA cultured lymphocytes were added as the experimental group. At 48 h after B1 asRNA treatment, a total of 1% formaldehyde was added into thelymphocytes for 30 min at room temperature to produce cross-linked protein and the DNA. Then glycine was used to terminate the effect of formaldehyde. The lymphocytes were re-suspended in 1 x Buffer A, incubated on ice for 10 min, and then centrifuged at 3000 rpm at 4 ˚C for 5 min to precipitate the nuclei. The nuclei were re-suspended and micrococcus nuclease was added to digest the DNA into fragments of 150 to 900 bp. Digestion was terminated by adding 0.5 mol/l EDTA. The nuclei werethen treated for 5 min using ultrasonic cell disruptor (Sonics & Materials, Inc., USA) and centrifuged at 10,000 rpm at 4 ˚C for 10 min. The supernatant was used for the next experiment. Rabbit anti-ZFP92 antibody (Beijing Biossbio technology Co. Ltd., China) was added to the sample and incubated at 4 ˚C overnight (rabbit IgG was used as negative control). To adsorb the complex of ZFP92 protein and ZFP92 antibody, protein G magnetic beads were added; If the ZFP92 protein binds to the DNA, then the DNA is also attached to the protein G magnetic beads. The absorbed DNAs were then eluted from the protein G magnetic beads. The DNAs were purified. qPCR was used to analyze ChIP enrichment efficiency. The following formula was used to calculate the ChIP enrichment efficiency: 2% x 2 ^(C[T]Input sample−C[T]IP Sample)^. Table 2 shows the primers for ChIP-qPCR.

According to the requirements of the ChIP kit manual, a negative control (normal rabbit IgG control) was set up. In this experiment, the negative control was the qPCR CT value obtained by replacing the rabbit anti-ZFP92 protein IgG with normal rabbit IgG, which reflected the background adsorption (nonspecific adsorption). All results of CT values in this experiment subtracted the CT value of negative control to eliminate the influence of nonspecific adsorption.

**Table 2:** ChIP-qPCR primer used for amplification.

Target	Sequences	Product length (bp)
Nanog#1	Forward: 5ʹ-ATTTCTTCTTCCATGCTTAGACGGCTGAG-3ʹ	150
	Reverse: 5ʹ-CTACCACCATGCCCAATTTAAGGAGTGTTT-3ʹ	
Nanog#2	Forward: 5ʹ-CCAGGTTTCCCAATGTGAAGAGCAAGCAA-3ʹ	170
	Reverse: 5ʹ-TGGCGATCTCTAGTGGGAAGTTTCAGGTCA-3ʹ	
Nanog#3	Forward: 5ʹ-GAGGATGCCCCCTAAGCTTTCCCTCCC-3ʹ	175
	Reverse: 5ʹ-CCTCCTACCCTACCCACCCCCTATTCTCCC-3ʹ	
Nanog#4	Forward: 5ʹ-CTCTTTCTGTGGGAAGGCTGCGGCTCACTT-3ʹ	165
	Reverse: 5ʹ-CATGTCAGTGTGATGGCGAGGGAAGGGA-3ʹ	
Nanog#5	Forward: 5ʹ-GCGGGTGTCCTTATCACTCTTCTGGAAA-3ʹ	182
	Reverse: 5ʹ-TCCAAGCTAGGATGTTAGGTCTCCCTGCTA-3ʹ	
Oct4#1	Forward: 5ʹ-GTGGTGGAGAGTGCTGTCTAGGCCTTAG-3ʹ	213
	Reverse: 5ʹ-AGCAGATTAAGGAAGGGCTAGGACGAGAG-3ʹ	
Oct4#2	Forward: 5ʹ-TGCTCTGGGCTTTTTGAGGCTGTGTGATT-3ʹ	249
	Reverse: 5ʹ-TGGCGGAAAGACACTAAGGAGACGGGATT-3ʹ	
Oct4#3	Forward: 5′-GGGGAGGGGTGGGTGACGAGGATGA-3ʹ	198
	Reverse: 5ʹ-TACTCAACCCTTGAATGGGCCAGGATGGCT-3ʹ	
Oct4#4	Forward: 5ʹ-GGGGGTGGTTAGTGTCTAATCTACCAACCT-3ʹ	209
	Reverse: 5ʹ-ACCCAGTATTTCAGCCCATGTCCAA-3ʹ	
Sox2	Forward: 5ʹ-GCCTTTGCACCCTTTGGATG-3ʹ	177
	Reverse: 5ʹ-GGTTCCCAAACACGAGTCCT-3ʹ	
Klf4	Forward: 5ʹ-GCCGCTCTCTTTCATAGCAG-3ʹ	202
	Reverse: 5ʹ-ATTATCCGCGTGACTCATCC-3ʹ	
Myc	Forward: 5ʹ-GAGAGAGGTGGGGAAGGGAGAAAG-3ʹ	135
	Reverse: 5ʹ-AGTGAGGCGAGTCGGACCCGGCA-3ʹ	

### Statistical analysis

All experiments were performed atleast in triplicates and quantitative data were presented as the mean ± standard deviation. GraphPad Prism software (version 6.01; GraphPad; Dotmatics) was used for statistical analysis. Differences among multiple groups were calculatedusing one‑way ANOVA followed by post‑hoc Bonferroni’s correction. * *P* < 0.05, ** *P* < 0.01, ***P < 0.001, indicated statistical significance.

## Results

### B1 AsRNA enhances the proliferative rate and decreases the apoptosis of senescent lymphocytes

**Fig. 1:**
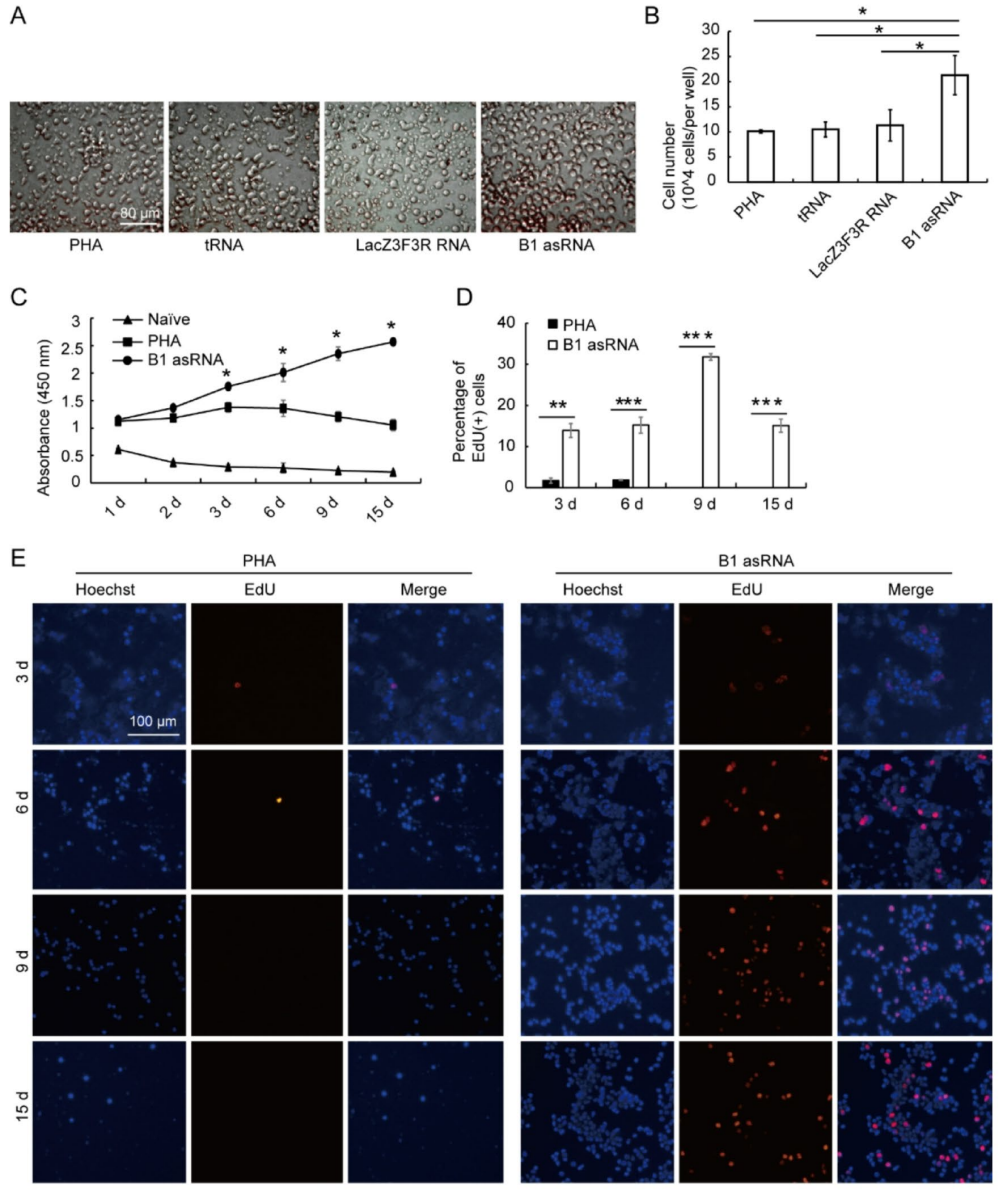

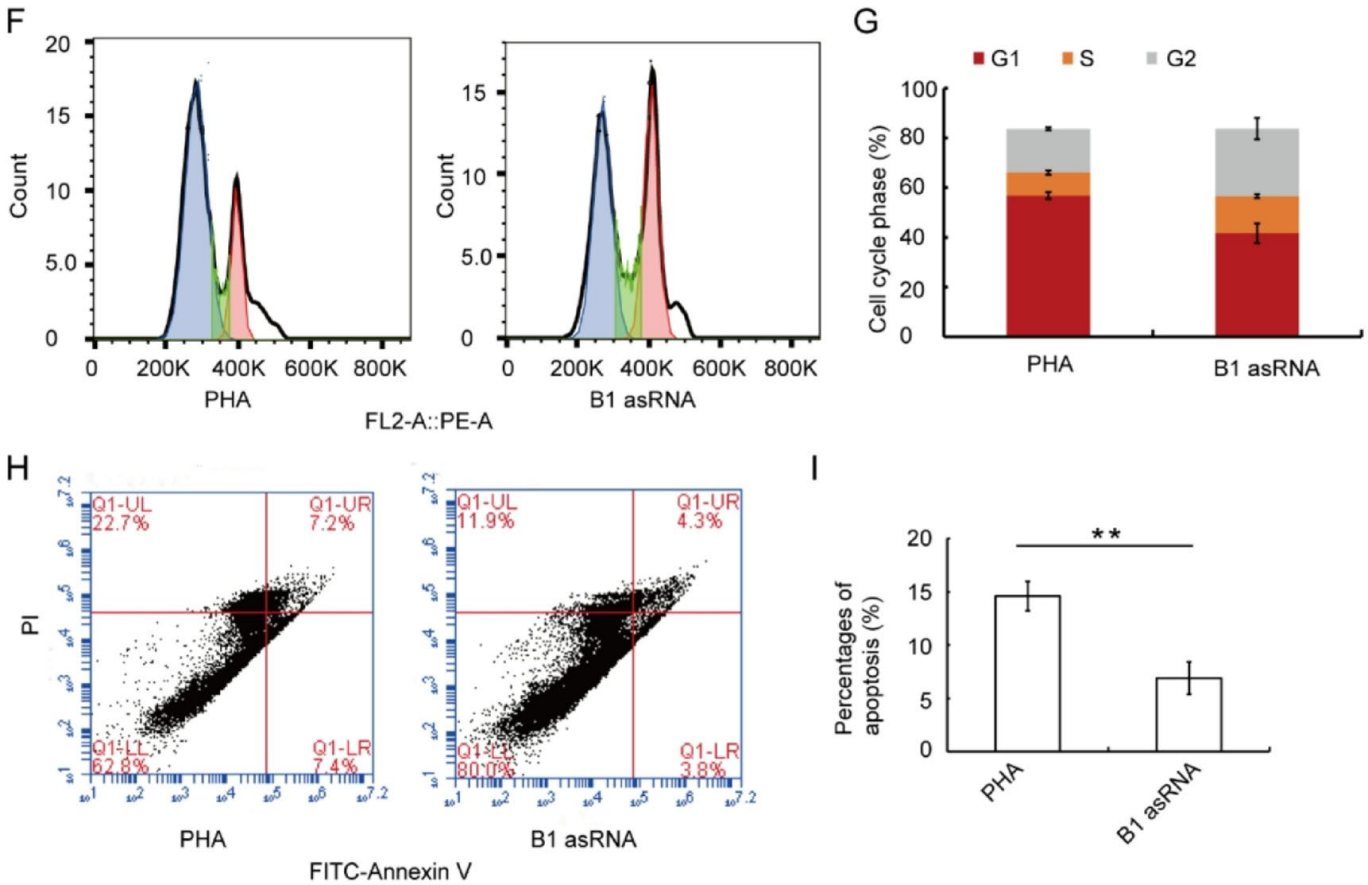
B1 asRNA enhances the proliferative rate and decreases the apoptosis of senescent lymphocytes. (**A**) Representative images of lymphocytes in different RNA group at 15 days after RNA transfection (magnification 10 × 10). (**B**) The cell number in each group at 15 days after different RNA transfection. The cell numbers are shown as mean ± SD of the five independent experiments, statistical differences were calculated using one‑way ANOVA with Bonferroni). *P < 0.05. (**C**) At different time points (1 day, 2 days, 3 days, 6 days, 9 days, 15 days), the viability of lymphocytes transfected with B1 asRNA was measured by CCK-8 (mean ± SD of three independent experiments). The cell viability of lymphocytes treated with B1 asRNA was significantly higher than that of the PHA group and the Naïve groups. *P < 0.05. B1 asRNA: the lymphocytes transfected with B1 asRNA on the basis on PHA stimulation; PHA: PHA-stimulated spleen lymphocytes; Naïve: the lymphocytes that are not stimulated with any substance. (**D**) EdU experiment results showed that the percentage of EdU positive cells in the B1 asRNA group was significantly higher than that in the PHA group (means of three independent experiments, statistical differences were calculated using one‑way ANOVA with Bonferroni). (**E**) EdU staining representative images of lymphocytes in the B1 asRNA group and the PHA control group (Magnification 10 × 10). Hoechst: results of nuclei Hoechst staining; EdU: EdU staining results; Merge: The results of overlapping Hoechst and EdU staining. (**F**) Detection of cell cycle of lymphocyte by flow cytometry. The peaks of S phase and G2/M phase in the B1 asRNA group were significantly higher than those in the PHA group. (**G**) Calculated from the results in (**F**), the proportion of lymphocytes in each phase of the cell cycle in the two groups (mean ±SD of three independent experiments). *P < 0.05. (**H**) Flow cytometry was used to detect apoptosis of lymphocytes in PHA group and B1 asRNA group. UL: Debris and damaged cells; UR: late apoptotic and dead cell; LL: normal living cells; LR: early apoptotic cells. (**I**) The percentages of early and late apoptosis of spleen lymphocytes in the two groups were calculated from the results in (**H**) (mean ± SD of three independent experiments). **P < 0.01.

After stimulating the growth of spleen lymphocytes from aged mice with PHA, B1 asRNA, tRNA or LacZ3F3R RNA were transfected into lymphocytes by calcium phosphate transfection (CPT) method, respectively, based on PHA stimulation. After transfection for 15 days, the growth of the cells was observed. Figure 1A and B show that the number of cells in the B1 asRNA treated group was significantly higher than that in the PHA, tRNA and LacZ3F3R RNA groups. As shown in Fig. 1C, with the increase of culture time, the lymphocyte viability in the B1 asRNA group increased, whereas the viability in the PHA stimulated group (PHA group) decreased after 6 d. In the Naïve group (without any treatment), the proliferative viability of cells gradually decreased with the extension of culture time.

The proliferation ability of lymphocytes was also detected using the EdU incorporation method at 3 d, 6 d, 9 d and 15d post transfection with 2.8% B1 asRNA. Figure 1D shows that the proliferation ability of lymphocytes in the B1 asRNA group was significantly higher than that in the PHA group and with the increase of transfection time, the amount of red fluorescence in lymphocytes in the B1 asRNA group increased, reaching a maximum value on day 9. Figure 1E shows representative images of EdU staining at 3 d, 6 d, 9 d and 15 d after B1 asRNA treatment. These results indicate that B1 asRNA increases the proliferation rate of lymphocytes as treatment time increases.

As shown in Fig. 1F, lymphocytes in the PHA group were mainly in the G1 phase, whereas lymphocytes in the B1 asRNA treatment group were significantly increased in the S phase and G2/M phase. The proportions of G1, S and G2 phases in lymphocytes of the two groups calculated according to the results of flow cytometry in Fig. 1F, indicating that the percentages of S and G2 phases in the B1 asRNA group were significantly higher than those in the PHA group (t values were 3.842 and 8.429, respectively, *P* < 0.05) (Fig. 1G). These results indicate that the proportion of S phase and G2 cell cycle in lymphocytes in the B1 asRNA group was significantly increasedcompared with the PHA group, and the proliferation ability of lymphocytes was significantly enhanced.

Figure 1H show that the apoptosis rate of lymphocytes in the B1 asRNA group was significantly lower than that in the PHA group, and that the percentage of apoptotic cells in the B1 asRNA group was significantly lower than that in the PHA group (t = 7.791, *P* < 0.05) (Fig. 1I), indicating that in the B1 asRNA inhibited the apoptosis of lymphocytes.

### B1 AsRNA enhances the tumor killing function of lymphocytes in vitro and in vivo

**Fig. 2:**
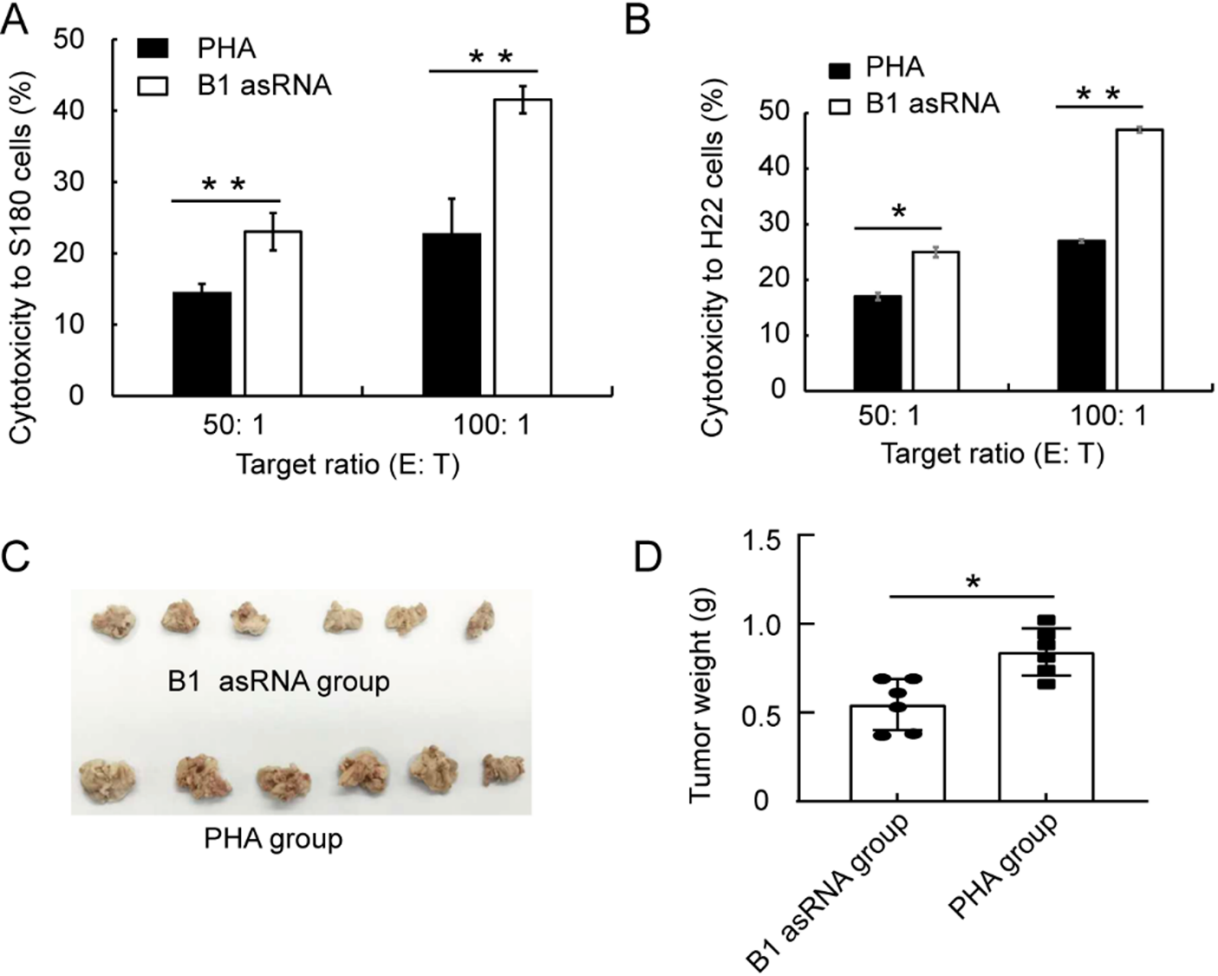
B1 asRNA enhances the tumor killing function of lymphocytes in vitro and in vivo. (**A**) LDH release assay shows that the ability of killing S180 cells of lymphocytes in the B1 asRNA group was higher than that in the PHA group (means of three independent experiments, statistical differences were calculated using one‑way ANOVA with Bonferroni). *P < 0.05. (**B**) LDH release assay shows that the ability of killing H22 cells of lymphocytes in the B1 asRNA group was higher than that in the PHA group (means of three independent experiments, statistical differences were calculated using one‑way ANOVA with Bonferroni). *P < 0.05. (**C**) The comparison of the two groups of masses in vivo experiments. (**D**) In vivo experiments show that tumor weight of the B1 asRNA group was significantly lower than that of the PHA group (mean ± SD of three independent experiments). *P < 0.05.

Figure 2A shows that the ability of killing S180 cells of lymphocytes in the B1 asRNA group was higher than that in the PHA group (*P* < 0.05) (the results of 15 d). In the same way, we found that the ability of killing H22 cells of lymphocytes in the B1 asRNA group was higher than that in the PHA group (*P* < 0.05) (Fig. 2B, the results of 15 d). These results indicated that B1 asRNA enhances the tumor killing function of lymphocytes in vitro. We aslo detected the the lymphocyte killing function at 6 d after B1 asRNA treatment. We found that ability of killing S180 cells of B1 asRNA treated lymphocytes at 6 d also was significantly higher than that in PHA group (data not shown).

The results of in vivo experiment showed that the mass of B1 asRNA group was significantly smaller than that of PHA group. Figure 2C shows the comparison of the two groups of masses, and Fig. 2D shows that the tumor weight of the B1 asRNA group was significantly lower than that of the PHA group.

### B1 AsRNA down-regulates expression of senescence related genes

**Fig. 3:**
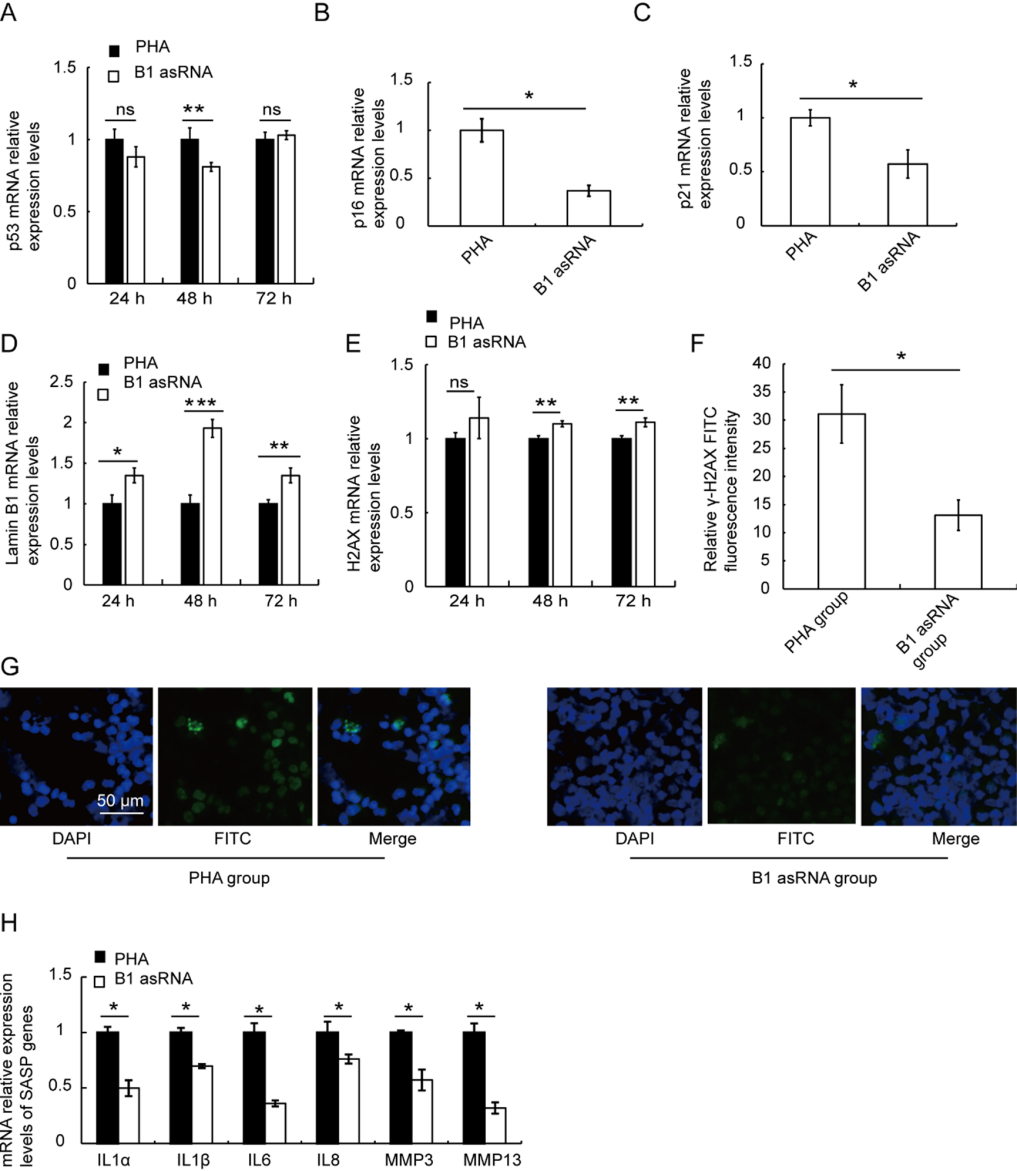
Effects of B1 asRNA on the expression of senescence related genes. (**A**) Effect of B1 asRNA treatment on the mRNA expression levels of the p53 gene. Compared with the PHA group, the expression of p53 mRNA in the B1 asRNA group was significantly down-regulated at 48 h of treatment (mean ±SD of three independent experiments). **P < 0.01. (**B**) B1 asRNA down-regulated the expression level of p16^Ink4a^ gene at 48 h after treatment (mean ±SD of three independent experiments).* *P* < 0.05. (**C**) B1 asRNA down-regulated the expression level of p21 gene at 48 h after treatment (mean ±SD of three independent experiments).* *P* < 0.05. (**D**) Effect of B1 asRNA treatment on the mRNA expression levels of the Lamin B1 gene. Lamin B1 mRNA expression was significantly up-regulated at 24 h, 48 h and 72 h of treatment (mean ± SD of three independent experiments).* *P* < 0.05, ** *P* < 0.01, *** *P* < 0.001. (E) Effect of B1 asRNA treatment on the mRNA expression levels of the H_**2**_AX gene. Compared with the PHA group, the expression of H_**2**_AX mRNA was significantly up-regulated at 48 h and 72 h of treatment (mean ±SD of three independent experiments). *: *P* < 0.05. (**F**) B1 asRNA down-regulated the protein level of γ-H2AX at 48 h after treatment. (**G**) Representative image of γ-H2AX protein detection by IF (magnification 25 × 10). (**H**) Effect of B1 asRNA treatment on the mRNA expression levels of the SASP genes. The mRNA expression levels were significantly down-regulated at 48 h after B1 asRNA treatment (mean ±SD of three independent experiments). *P < 0.05.

The p53 protein is involved in DNA damage response and is also a key regulator of the cell cycle^24^. The lymphocytes treated with PHA were used as the control group (PHA group), and lymphocytes treated with B1 asRNA based on PHA stimulation were used as the experimental group (B1 asRNA group). Our findings show that the p53 mRNA level in the B1 asRNA group was down-regulated after 48 h of treatment with statistical significance compared with those of the PHA group, (*t* = 7.416, *P* < 0.05) (Fig. 5A). In addition, Fig. 3B and C show that B1 asRNA significantly reduced the expression levels of aging-related genes p16 and p21 at 48 h of treatment.

Lamin B1 is the main component of the nuclear layer, and its down-regulation is another important feature of cell senescence. Loss of Lamin B1 is associated with epigenetics and chromatin structure associated with aging^25^. Our findings indicate that compared with the PHA group, Lamin B1 mRNA expression was upregulated at 24 h, 48 h and 72 h with statistical significance after B1 asRNA treatment (*t*-values were 4.129, 10.03 and 5.699, respectively, *P* < 0.05) (Fig. 3D). H_2_AX mRNA expression levels were up-regulated 24 h, 48 h and 72 h after B1 asRNA treatment and had statistical significance at 48 h and 72 h compared with those of the PHA group (*t*-values were 5.096 and 4.715, respectively, *P* < 0.05) (Fig. 3E).

DNA double-strand breaks (DSBs) are extremely dangerous lesions with severe consequences for cell survival and the maintenance of genomic stability. In higher eukaryotic cells, DSBs in chromatin promptly initiate the phosphorylation of the histone H2A variant, H2AX, at Serine 139 to generate γ-H2AX. γ-H2AX is a typical marker of DNA damage response. The immunofluorescence (IF) microscopy results (Fig. 3F, G) shows that B1 asRNA significantly decreased the positive rate of γ-HAX in senescent lymphocytes.

Senescence-associated secretory phenotype (SASP) is marked by senescent cells that secrete a wide range of biomolecules. These include chemokines, inflammatory cytokines, proteases, and growth factors. SASP is key to cellular senescence^26^. Figure 3H shows that B1 asRNA significantly down-regulated the expression level of the SASP genes including IL1α, IL1β, IL6, IL8, MMP3 and MMP13.

### B1 AsRNA up-regulates the expression of transcription factor genes

**Fig. 4:**
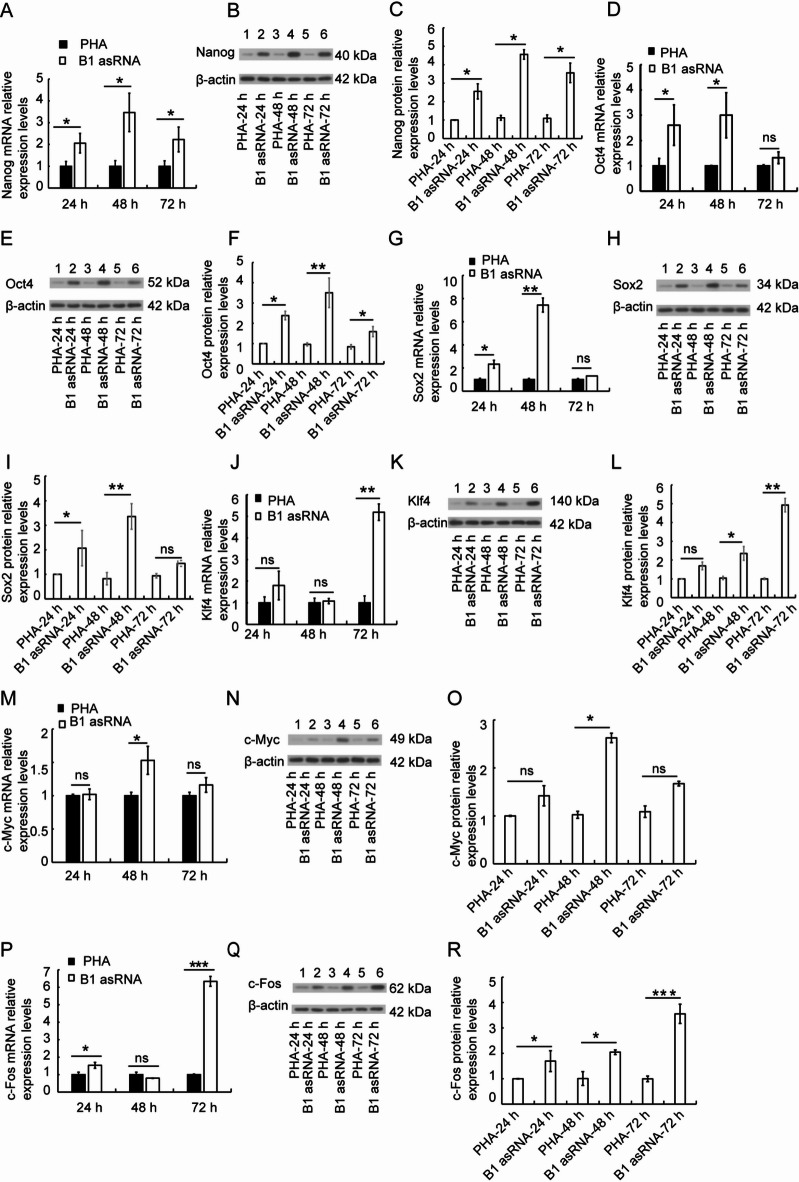
The effects of B1 asRNA on the expression of transcription factor genes. RT-qPCR was used to detect the mRNA expression of Nanog, Oct4, Sox2, Klf4, c-Myc and c-FOS in lymphocytes of PHA group and B1 asRNA group at 24 h, 48 h and 72 h in vitro. The mRNA expressions of Nanog (**A**), Oct4 (**D**), Sox2 (**G**), Klf4 (**J**), c-Myc (**M**) and c-Fos (**P**) were up-regulated 24 h, 48 h and 72 h after B1 asRNA treatment (mean ± SD of three independent experiments). *P < 0.05, **P < 0.01, ***P < 0.001. Western blotting was used to detect the protein expression levels of Nanog, Oct4, Sox2, Klf4, c-Myc and c-FOS in lymphocytes of PHA group and B1 asRNA group at 24 h, 48 h and 72 h in vitro. The protein expression levels of Nanog (**B–C**), Oct4 (**E–F**), Sox2 (**H**, **I**), Klf4 (**K**,** L**), c-Myc (**N**, **O**) and c-Fos (**Q**,** R**) were up-regulated after B1 asRNA treatment. The protein relative expression levels were means ±SD of three independent experiments). *P < 0.05, **P < 0.01.

Transcription factors Oct4, Sox2, Klf4 and Myc can reprogram somatic cells into induced pluripotent cells and regulate many genes related to cell aging. When we tested the mRNA expression level of transcription factors Nanog, Oct4, Sox2, Klf4 and Myc using RT-qPCR, our results showed that the mRNA expressions of Nanog (Fig. 4A), Oct4 (Fig. 4D), Sox2 (Fig. 4G), Klf4 (Fig. 4J) and Myc (Fig. 4M) were up-regulated at 24 h, 48 h and 72 h. The mRNA levels of other genes except Klf4 gene were the highest at 48 h, and these results were statistically significant (*P* < 0.05).

c-Fos is another important transcription factor. We found that compared with the PHA control group, c-Fos mRNA expression was up-regulated at 24 h and 72 h of treatment, with statistical significance (*t-*values were 4.099 and 31.86, respectively,*P* < 0.05) (Fig. 4P).

Western blotting results show that B1 asRNA up-regulated the expression levels of proteins Nanog (Fig. 4B-C), Oct4 (Fig. 4E-F), Sox2 (Fig. 4H-I), Klf4 (Fig. 4K-L), Myc (Fig. 4N-O) and c-Fos (Fig. 4Q-R).

The original gels of Western blotting of Nanog, Oct4, Sox2, Klf4, c-Myc and c-Fos are presented in Supplementary Fig. 1.

### ZFP92 protein is involved in the role of B1 AsRNA

**Fig. 5:**
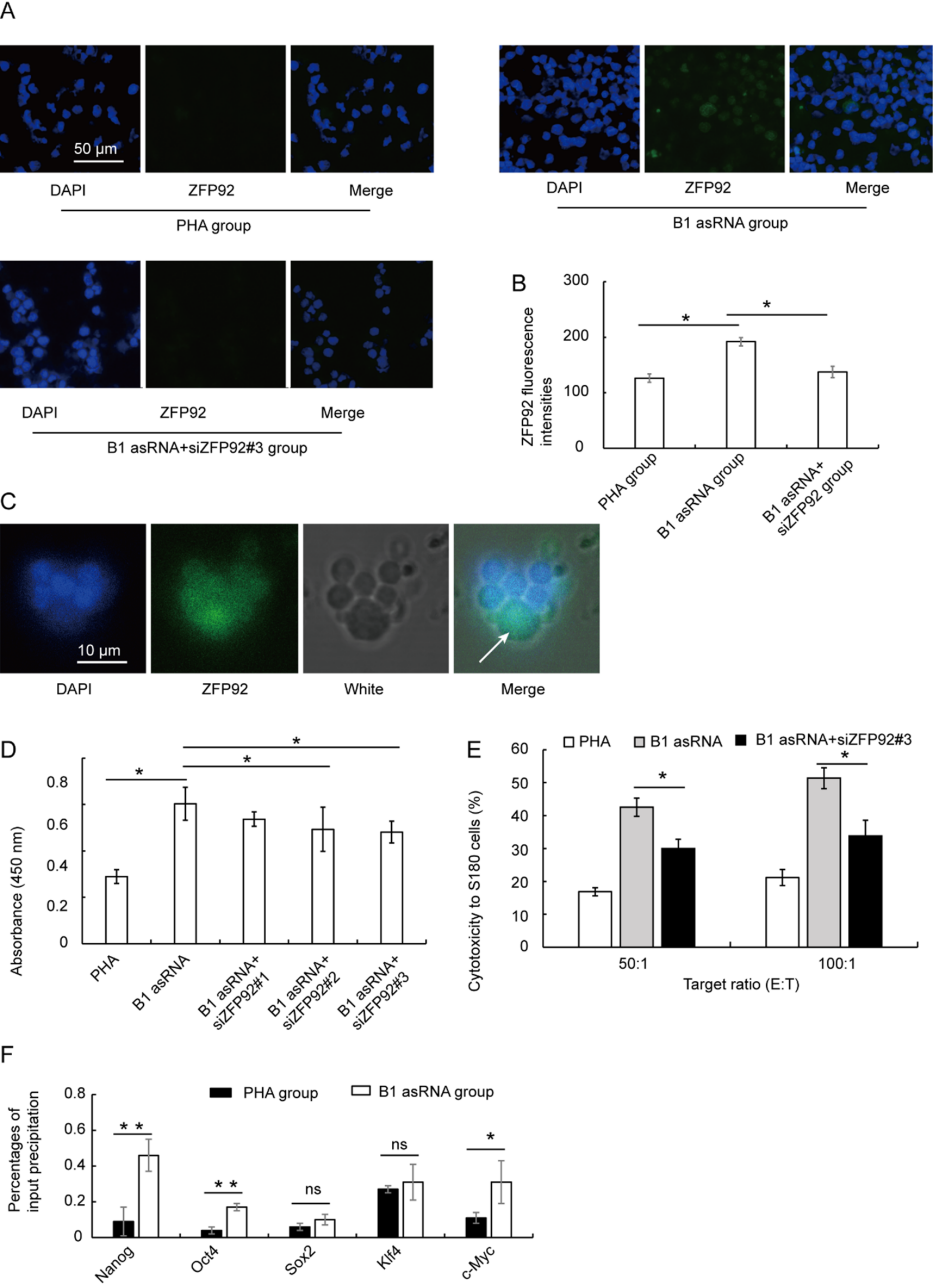
Effects of B1 asRNA on ZFP92 protein expression. (**A**) Representative images of ZFP92 IF microscopy (magnification 25 × 10). The fluorescence intensity of ZFP92 in B1asRNA culture group was higher than that in the PHA culture group and siZFP92#3 significantly reduced the expression level of ZFP92 protein induced by B1asRNA. (**B**) The fluorescence intensity of each group was calculated by Image J software. The fluorescence intensity of ZFP92 in B1 asRNA group was significantly higher than that in PHA group (mean ± SD of three independent experiments). **P < 0.01. (**C**) Staining of euchromatin and heterochromatin regions in the nucleus of lymphocytes observed by laser confocal microscopy (magnification 100 × 10). The DAPI-labeled nuclear regions and FITC-labeled ZFP92 protein regions did not overlap. ZFP92 protein fluorescence in large lymphoblastoid cells was stronger than that in small lymphocytes, but its DAPI staining was lighter than in small lymphocytes. DAPI: Results of DAPI staining of nuclei; ZFP92: ZFP92 staining results; Merge: Results of overlapping DAPI and ZFP92 stains; White: White light. The arrow indicates the large lymphocyte. (**D**) CCK8 assay showed that the cell proliferation activity significantly decreased when the lymphocytes were treated with ZFP92 siRNA (mean ± SD of three independent experiments). *P < 0.05. siZFP92#3 had the strongest effect in reducing the proliferation activity of lymphocytes. Therefore, siZFP92#3 was used in the experiment shown in (**A**), and it was also continued to be used in the experiments of (**E**). (**E**) LDH release assay shows that siZFP92#3 significantly decreased the ability of killing S180 cells of lymphocytes (means of three independent experiments, statistical differences were calculated using one‑way ANOVA with Bonferroni). *P < 0.05. (**F**) Effects of mouse B1 asRNA on DNA expression of transcription factors in spleen lymphocytes. ChIP-qPCR was used to detect the effect of B1 asRNA treatment on the binding ability of ZFP92 protein to transcription factors (Nanog, Oct4, Sox2, Klf4, c-Myc) in spleen lymphocytes from aged mice. Nanog, Oct4, Sox2, Klf4 and Myc DNA enrichment efficiency of ZFP92 protein in B1 asRNA treated group was higher than that in conventional culture group (PHA group) (mean ±SD of three independent experiments). *P < 0.05, **P < 0.01.

The ZFP92 gene is located on the X chromosome, and its primary role in mice is to regulate the activity of B1/Alu SINE elements and regulate the activity of surrounding genomic entities by binding to the ZFP92 binding motif shared by 28bp^27^. To investigate the effect of B1 asRNA on ZFP92 protein expression in spleen lymphocytes, conventional cultured lymphocytes were used as the control group, and B1 asRNA cultured lymphocytes were added as the experimental group. Our results show that the fluorescence intensity of ZFP92 in the B1 asRNA culture group is higher than that in the conventional culture group and siRNA-ZFP92 (siZFP92#3) could significantly inhibit the expression of ZFP92 at the protein level (Fig. 5A, B). In addition, staining of the ZFP92 protein in spleen lymphocytes of aged mice under confocal microscopeshowed that euchromatin and heterochromatin regions in large lymphocytes do not overlap the ZFP92 protein regions labeled by DAPI and FITC (Fig. 5C), indicating that ZFP92 protein in heterochromatin region is less than in theeuchromatin region. The euchromatin and heterochromatin regions of the small lymphocytes displayedmore blue stain and less green stain asobserved by confocal microscope. These results suggest an increased binding of the ZFP92 protein to DNA in lymphocytes treated with B1 asRNA and located in the euchromatin region of the nucleus.

To explore whether the proliferative and cytotoxic effects of B1 asRNA are indeed ZFP92-dependent, we used CCK-8 assay and LDH release assay to detect detect the proliferative ability and the killing function of lymphocytes on S180 cells. Figure 5D shows that the cell proliferation activity significantly decreased when the lymphocytes were treated with ZFP92 siRNA. Figure 5E shows that ZFP92 siRNA (siZFP92#3) significantly decreased the ability of killing S180 cells of lymphocytes. These results suggested that the proliferative and cytotoxic effects of B1 asRNA are indeed ZFP92‐dependent.

SINE-KRAB-ZFP interaction is a key regulator of chromatin accessibility of elements. The expression of transcription factors (Nanog, Oct4, Sox2, Klf4, Myc) binding to ZFP92 protein in lymphocytes was detected by ChIP-qPCR. Our results show that, compared with the PHA control group, the binding ability of Nanog, Oct4, Sox2, Klf4, Myc and ZFP92 proteins in the B1 asRNA treatment group was increased, and the binding ability of Nanog, Oct4 and c-Myc to ZFP92 proteins was statistically significant (*P* < 0.05) (Fig. 5F). These findings indicate that B1 asRNA can recruit ZFP92 proteins to bind to Nanog, Oct4, Sox2, Klf4, c-Myc genes.

## Discussion

Aging is a biological process that leads to a decline in vitality and health through the accumulation of many different molecular and cellular damages^28^. Aging also increases the risk of cancer and other diseases. Immune cells are a key factor in the treatment of diseases such as tumors^29^, and lymphocytes are the basic components of the immune system and are widely distributed in the organisms. T lymphocytes and B lymphocytes can be activated by antigen stimulation to occur proliferation, division and specific immune responses^30^.

In this study, PHA treatment and B1 asRNA treatment methods were established to culture spleen lymphocytes from aged mice. We found that the number of lymphocytes was the highest and the proliferation viability of lymphocytes was the strongest under 2.8% B1 asRNA treatment. 2.8% B1 asRNA increased the number of lymphocytes in the S and G2/M phases of the cell cycle and reduced apoptosis of lymphocytes. In addition, B1 asRNA also improved lymphocyte ability to kill S180 and H22 tumor cells.

Our previous work demonstrated that B1 asRNA has anti-fatigue and antioxidant activities in naturally aged BALB/c mice, can reduce the accumulation of ROS in aged mouse cells, and can regulate the expression level of age-related genes^7^. In this study, the effect of B1 asRNA on the expression of age-related genes in spleen lymphocytes from aged mice was further studied. We found that B1 asRNA can significantly reduce the mRNA expression levels of p53, p16, p21 and SASP genes. The p53 tumor suppressor protein is involved in DNA damage response and is also a key regulator of the cell cycle^31^. γ-H_2_AX is a typical marker of DNA damage response and participates in identification and repair of DNA double-stranded break and maintains genomic stability^32^. The down-regulation of Lamin B1 is also an important feature of cell senescence^25^. Based on our results, B1 asRNA increased the mRNA expression levels of Lamin B1 and H2AX genes and reduced the protein level of γ-H_2_AX. These results indicated that B1 asRNA can regulate the expression of age-related genes.

Moreover, we found that B1 asRNA treatment improved the lymphocyte activity and killing tumor function and enhanced the proportion of the large lymphocytes. The results of confocal microscopy show that the large lymphocytes had increased cell volume, more cytoplasm, and looser chromatin (Fig. 5C, as indicated by the arrow). We also observed deep DAPI staining and light FITC-ZFP92 staining in the nuclei of small lymphocytes, whereas the large lymphocyte nuclei showed stronger FITC-ZFP92 staining and lighter DAPI staining (Fig. 5C). In the large lymphocyte nuclei, FITC-ZFP92 staining was stronger in the DAPI-light region and lighter in the DAPI-deep region, indicating more ZFP92 protein aggregation in the euchromatin region. It has been reported that transcribed regions belong to euchromatin regions and gene silencing regions belong to heterochromatin regions^33,34^. Our results suggest that B1 asRNA was able to activate ZFP92 protein expression and the expressed ZFP92 protein is concentrated in the euchromatin region.

ZFP92 has been shown to regulate the activity of genes surrounding SINE elements by binding to B1/Alu elements in mice^27^. Our results found that B1 asRNA can promote the expression levels of transcription factor Nanog, Oct4, Sox2, Klf4, Myc and c-Fos genes (Fig. 4). By searching the mouse Nanog, Oct4, Sox2, Klf4 and Myc genes and their upstream and downstream 10,000 bp repeat sequences (Supplementary Fig. 2) in a UCSC browser, the number of B1 SINE elements in the repeat sequences was counted (Table 3). We found the largest number of B1 elements inside and flanking the Nanog gene. Because ZFP92 is a transcription factor that binds B1 elements, we proposed that regulating the expression level of transcription factor genes induced by ZFP92 protein may be related to B1 element number ingene internal and flanking sequences. The results of ChIP-qPCR showed that the content of the Nanog gene in the ChIP precipitation in B1 asRNA group increased significantly, indicating that the precipitation caused by ZFP92 protein contains the highest content of the Nanog gene DNA. These results indicate that B1 asRNA treatment up-regulates the expression of transcription factor genes and increases the copy number of transcription factor genes in anti-ZFP92-ChIP precipitation. There also appears to be a correlation between the number of B1 elements (in gene and its flanking) and the maintenance time of up-regulating the expression of transcription factor genes during the process of B1 asRNA regulating gene expression.

**Table 3:** B1 element number in *Nanog*, *Oct4*, *Sox2*, *Klf4* and *c-Myc* genes.

Gene	B1 element number		
	Upstream 10,000 bp	Gene sequence	Downstream 10,000 bp
Nanog	14	7	8
Oct4	12	0	6
Sox2	0	0	0
Klf4	5	0	7
c-Myc	5	0	6

B1 SINE: B1_Mus1, B1_Mus2B1_Mm, B1_Mur1, B1F, PB1D7, PB1D9, PB1D10; B1/Alufamilies; SINE class.

**Fig. 6:**
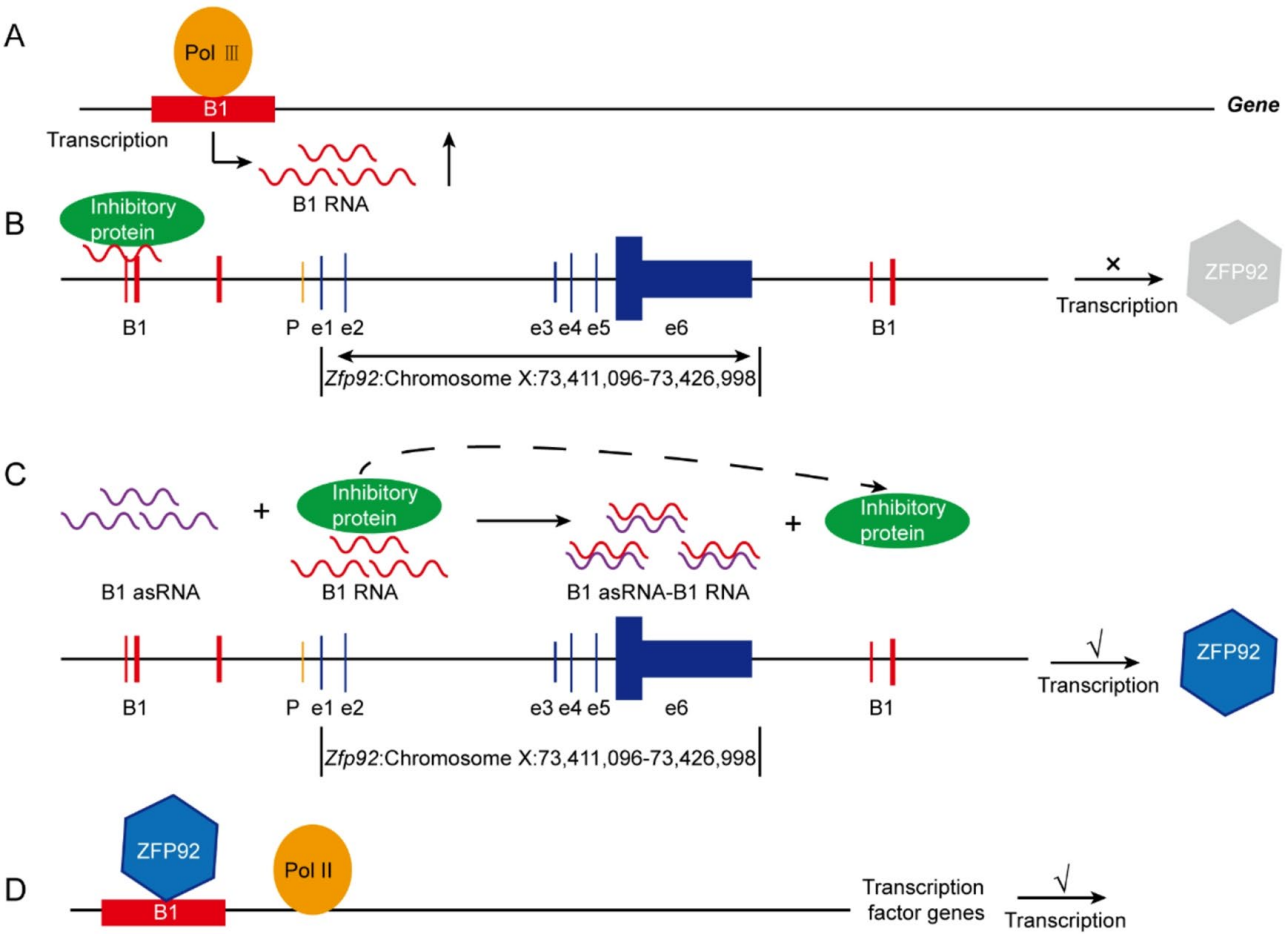
Potential mechanism of B1 asRNA action in enhancing the function of spleen lymphocytes from aged BALB/c mice. (**A**) In mice, B1 RNA level increases with thecell aging process due to transcription of B1 elements by RNA polymerase III. (**B**) Increased B1 RNA can bind to the inhibitory proteins to form RNA-protein complexes, which prevents the ZFP92 gene transcription. (**C**) When B1 asRNA was transfected into lymphocytes, B1 asRNA could bind to B1 RNA, which alleviated the inhibition effect of B1 RNA on ZFP92 gene transcription, thus increasing the expression of ZFP92 protein. (**D**) ZFP92 protein can bind to B1 elements in the transcription factor genes and its upstream and downstream sequences, resulting in increased expression of transcription factor genes.

In summary, B1 asRNA promotes the proliferation viability of spleen lymphocytes and improves the ability of the spleen lymphocytesto kill S180 tumor cells in vitro and in vivo. B1 asRNA can regulate the expression of age-related genes and up-regulate the mRNA and protein expressionlevels of transcription factors Nanog, Oct4, Sox2, Klf4 and Myc genes. We outline apossible molecular mechanism (Fig. 6) in which B1 asRNA enhances the function of spleen lymphocytes from aged BALB/c mice. Based on reported literature and our results of this study, B1 RNA expression is up-regulated during aging^35^. B1 asRNA binds with B1 RNA to alleviate the expression inhibition induced by B1 RNA on ZFP92 gene. The ZFP92 contains less B1 elements inside and flanking the gene sequence, B1 asRNA treatment significantly increased ZFP92 protein expression. We hypothesize that the B1 element in the ZFP92 gene may be located at a crucial regulatory position for transcription or B1 asRNA may have other ways of action except the binding with B1 RNA pathway, which needs further study.

## Conclusion

We concluded that B1 asRNA regulates the expression of senescence-related genes and transcription factor genes and that ZFP92 protein may play an important role in the process of B1 asRNA regulating gene expression. These studies suggest that B1 asRNA can enhance immune functions of senescent lymphocytes.

## Supplementary Information

Below is the link to the electronic supplementary material.

Supplementary Material 1

## Data Availability

The data generated in the present study may be requested from the corresponding author.

## Abbreviations

SINEs: Short interspersed nuclear elements
TEs: Transposable elements
KRAB-ZFP: Kruppel-associated box domain-containing zinc-finger proteins
PHA: Phytohemagglutinin
B1 asRNA: B1 antisense RNA
EdU: 5-Ethynyl-2ʹ-deoxyuridine
LDH: Lactate dehydrogenase
IOD: Integrated optical density
ChIP: Chromatin immunoprecipitation
CPT: Calcium phosphate transfection
SASP: Senescence-associated secretory phenotype
IF: Immunofluorescence
