# Supplementary material for: Role of B1 antisense RNA on the proliferation and killing tumor ability of aged mouse spleen lymphocytes
Source: Sci Rep. 2025 Nov 7;15:39138. doi: 10.1038/s41598-025-23139-z (PMC12594820; doi:10.1038/s41598-025-23139-z)
Supplement: Supplementary file 1 — Supplementary Material 1 [file 41598_2025_23139_MOESM1_ESM.docx]

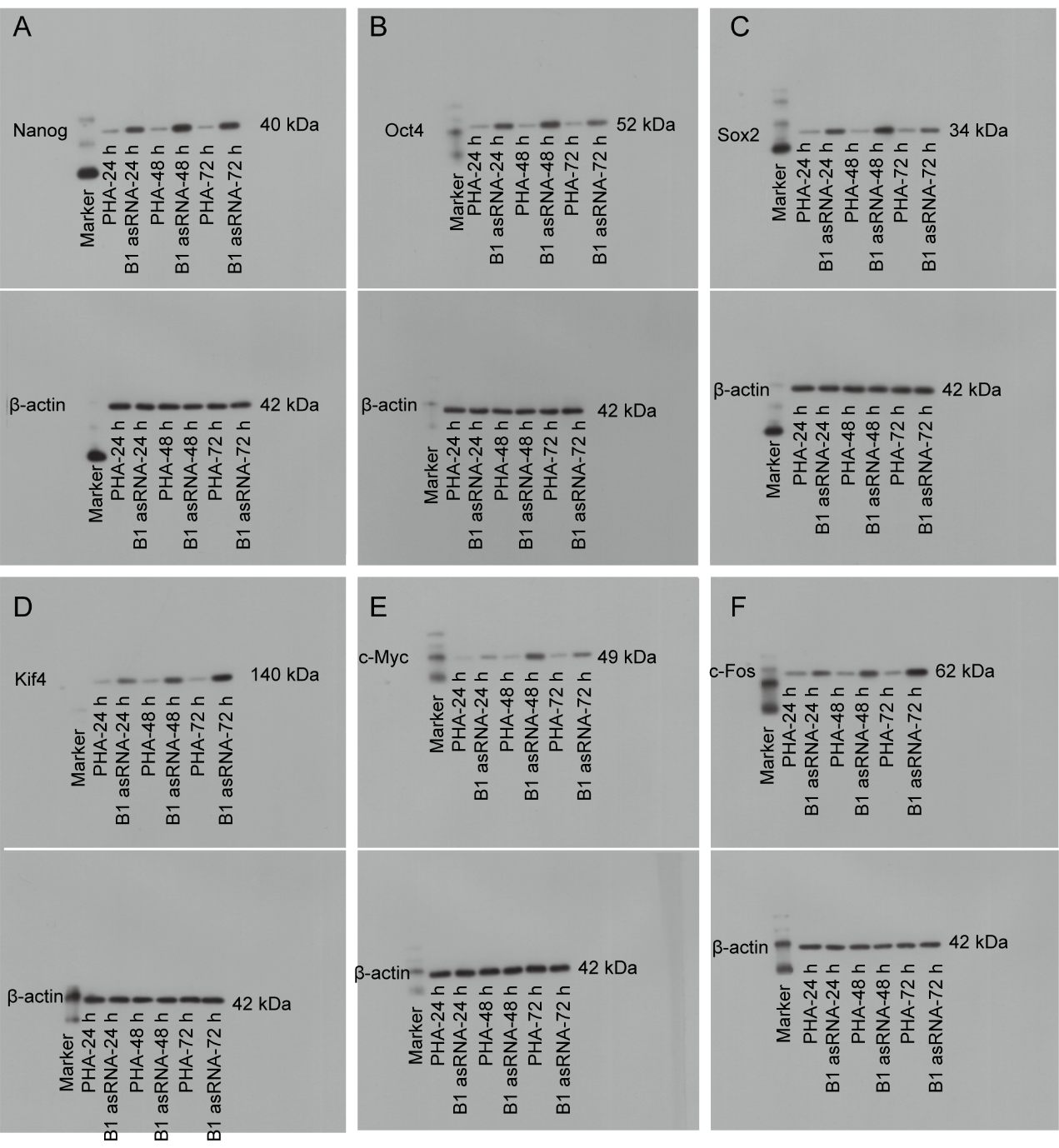


**Supplementary Fig.1**. The original gels of Western blotting. (**A**) The original gels of Nanog. (**B**) The original gels of Oct4. (**C**) The original gels of Sox-2. (**D**) The original gels of Kif4. (**E**) The original gels of c-Myc. (**F**) The original gels of c-Fos.


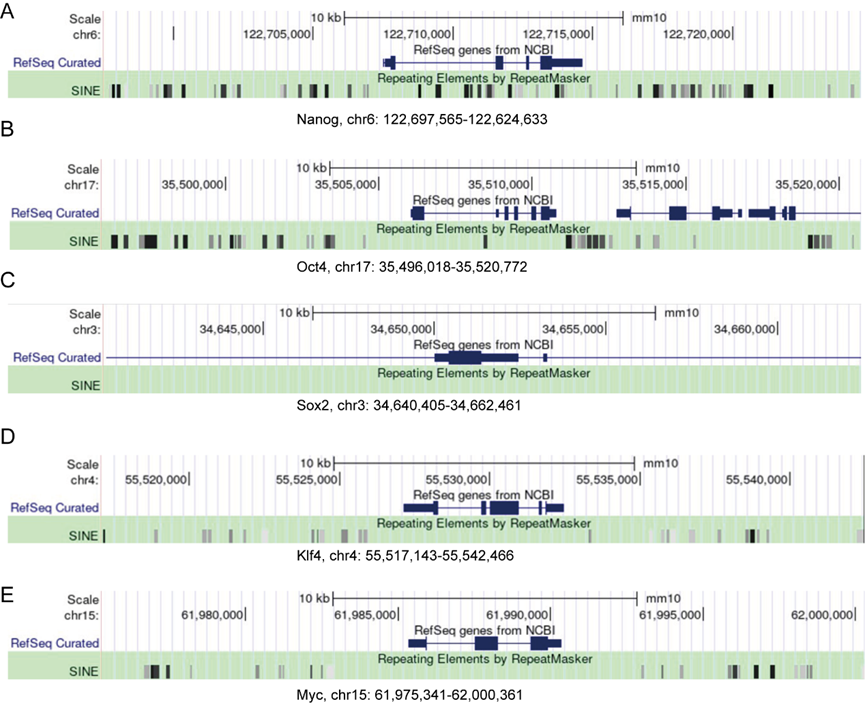


**Supplementary Fig. 2**. UCSC Genome Browser view of transcription factor gene regions on the mouse chromosomes (GRCm38/mm10). (**A**) UCSC Genome Browser view of Nanog gene*.* (**B**) UCSC Genome Browser view of Oct4 gene *.*(**C**) UCSC Genome Browser view of Sox2 gene. (**D**) UCSC Genome Browser view of Klf4 gene. (**E**) UCSC Genome Browser view of and Myc gene.
